# Intact plasma quantification of the large therapeutic lipopeptide bulevirtide

**DOI:** 10.1007/s00216-021-03384-7

**Published:** 2021-05-20

**Authors:** Max Sauter, Antje Blank, Felicitas Stoll, Natalie Lutz, Walter E. Haefeli, Jürgen Burhenne

**Affiliations:** 1 Department of Clinical Pharmacology and Pharmacoepidemiology, Heidelberg University Hospital, Im Neuenheimer Feld 410, 69120 Heidelberg, Germany; 2 German Center for Infection Research (DZIF), Heidelberg Partner Site, Im Neuenheimer Feld 410, 69120 Heidelberg, Germany

**Keywords:** Bulevirtide, Chronic hepatitis B and D, UPLC, Tandem mass spectrometry, Plasma, Bioanalysis

## Abstract

Bulevirtide is a first-in-class entry inhibitor of the hepatitis B and hepatitis delta virus blocking the sodium/bile acid co-transporter NTCP, and was recently approved for the treatment of hepatitis D as a priority medicine (prime) in an accelerated assessment by the European Medicines Agency. It is a very large lipopeptide comprising 47 amino acids in its sequence and a myristoylation at the N-terminus. For support of clinical development, we established highly sensitive plasma quantification assays using 100 μL of plasma, spanning concentrations of 0.1 to 100 ng/mL and 1 to 1000 ng/mL with the option to measure ten-fold diluted samples up to 10,000 ng/mL. Quantification was performed with UPLC-MS/MS measurements after extraction with protein precipitation. Both assays were fully validated according to the pertinent guidelines of the FDA and EMA, including incurred sample reanalyses and cross-validation using clinical study samples.

**Supplementary Information:**

The online version contains supplementary material available at 10.1007/s00216-021-03384-7.

**Figure:**
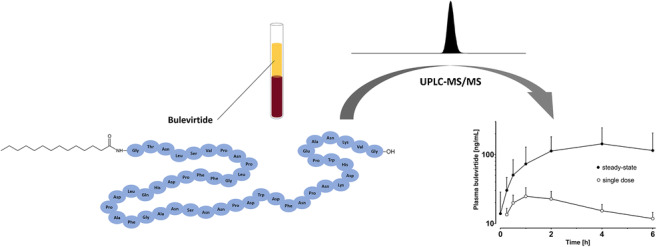
Graphical abstract

## Introduction

Bulevirtide (Hepcludex®), a first-in-class entry inhibitor of hepatitis B (HBV) and hepatitis delta virus (HDV), was recently approved for the treatment of the orphan disease chronic hepatitis D, where satisfying treatment alternatives are lacking (EU orphan drug designation number EU/3/15/1500, EU marketing authorization number EU/1/20/1446/001). The accelerated conditional approval as a priority medicine included the continuation of clinical trials to confirm efficacy and safety for chronic hepatitis D. Further studies also explore the efficacy in chronic hepatitis B.

Bulevirtide blocks the sodium taurocholate co-transporting polypeptide (NTCP; SLC10A1), the natural bile acid transporter, which at the same time is the high-affinity hepatic entry receptor of HBV and HDV [1]. Blocking NTCP prevents intrahepatic spreading of the virus. Clinically chronic HDV patients under bulevirtide showed markedly decreasing HDV titers and a normalization of the hepatic enzyme alanine aminotransferase, which is an important clinical surrogate for the disease outcome [2, 3].

Bulevirtide (formerly myrcludex B) is a very large lipopeptide, derived from the HBV large envelope protein (truncated pre-S1 domain), comprising 47 amino acids and a myristoylation at the N-terminus [4]. Its sequence is myristoyl-GTNLSVPNPLGFFPDHQLDPAFGANSNNPDWDFNPNKDHWPEANKVG-OH.

In support of the clinical development, we developed two related plasma quantification assays based on UPLC-MS/MS. However, sensitive MS/MS quantification of such large peptides is usually challenging due to the broad isotopic pattern and multiple differently charged species in electrospray ionization (ESI). Additionally, collision-induced dissociation (CID) induced through mobile protons at the amide bond nitrogen is generally non-selective for individual peptide bonds, which are the primary location of peptide fragmentation. The high number of peptide bonds in large peptides therefore results in a high number of generated product ions that additionally dilute signal intensity in selected reaction monitoring (SRM). These factors are anticipated to be particularly pronounced in quantification of intact bulevirtide; however, intact MS/MS quantification fosters highly selective determination.

Nowadays, peptide bioanalysis is starting to almost completely shift to MS/MS-methodologies due to superior dynamic range and especially selectivity (absence of cross-reactivity) in comparison to immunoassay [5, 6].

Because of the often-high potency of peptide therapeutics, the remaining challenge for their MS quantification is sufficiently high sensitivity. As a consequence, MS/MS assay development remains particularly challenging for CID-resistant peptides (which include in particular cyclized peptide drugs [7–10]) and for large peptides. For this purpose, methodologies to avoid CID of peptides in LC-MS quantification while concurrently ensuring sufficient selectivity have been established. These monitor the precursor directly, or a pseudo-transition (selected ion monitoring [SIM]) of the unfragmented precursor in MS/MS systems. For linear peptides, they particularly include ion mobility separations [10–14]. However, MS instruments providing these options are not broadly available, and the achievable sensitivities are often comparable or lower compared to standard MS/MS measurements [7]. Furthermore, we already successfully applied highly sensitive quantification via UPLC-MS/MS to other large therapeutic peptides [15, 16].

To allow regulatory bioanalysis of bulevirtide, we established a quantification for plasma bulevirtide consisting of two bioanalytical assays using UPLC-MS/MS quantification and extraction by protein precipitation with acetonitrile (ACN), in plasma samples of only 100 μL. The lower limit of quantification (LLOQ) was established at 0.1 ng/mL (19 pM) and the upper limit of quantification (ULOQ) at 1000 ng/mL with a low concentration assay ranging from 0.1 to 100 ng/mL and a high concentration assay spanning 1 to 1000 ng/mL. Additionally, dilution integrity of ten-fold diluted samples was established to extend the ULOQ to 10,000 ng/mL. Both assays were fully validated according to the guidelines for bioanalytical method validation of the FDA and EMA [17, 18]. Additionally, validity was assessed with clinical samples of an exploratory phase I trial in regard to incurred sample reanalysis and to cross-validate both assays to enable their interchangeable use for plasma bulevirtide quantifications. The wide concentration range of the plasma bulevirtide quantification will be required in current and further clinical trials to describe pharmacokinetic parameters of new emerging formulations or different treatment schedules as treatment may be optimized along time.

## Materials and methods

### Clinical study and plasma sample generation

Plasma samples containing bulevirtide for the purpose of cross-validation and incurred sample reanalysis evaluations were obtained from a monocentric exploratory phase I study which was carried out at the Clinical Research Unit of the Department of Clinical Pharmacology and Pharmacoepidemiology, which is certified according to EN ISO standard 9001. The trial was under the legal sponsorship of the Medical Faculty of the Heidelberg University. The trial followed the guideline of Good Clinical Practice, the ethical principles expressed in the Declaration of Helsinki, and all legal requirements for clinical trials in Germany. It was approved by the responsible Ethics Committee of the Medical Faculty of Heidelberg University (AFmo-670/2016) and the competent national authority (BfArM, EudraCT: 2017-003137-28). Prior to participation in any trial-related procedures, each participant provided written informed consent. Clinical results of the trial will be reported elsewhere. Pharmacokinetic profiles (pre-dose, 0.25, 0.5, 1, 2, 4, 6 h) were taken on the first day of treatment (single-dose pharmacokinetic assessment) and after 85 days of daily treatment with 5 mg subcutaneous bulevirtide (steady-state pharmacokinetic assessment). Blood samples were drawn into heparinized tubes (2.7 mL) immediately centrifuged at 2000*g* for 10 min at 4 °C, and plasma was stored at −20 °C until analysis.

### Drugs, chemicals, solvents, and materials

Bulevirtide acetate (91.4% peptide content) was obtained from MYR GmbH (Burgwedel, Germany) supplied by Chinese Peptide Company (Hangzhou, China). Isotopically labeled internal standard (IS) [^13^C_9_,^15^N]_3_-Phe^12,22,33^-bulevirtide (resulting mass difference 30 Da) was obtained from MYR GmbH supplied by Bachem AG (Bubendorf, Switzerland). An arium® mini (Sartorius, Göttingen, Germany) system was used for production of ultra-pure water. Methanol (MeOH), ACN, and formic acid (FA) of the highest available purity were purchased from BioSolve (Valkenswaard, The Netherlands). Blank Li-heparin plasma was obtained from donations of healthy volunteers.

### Standard solutions

Independent weighings of bulevirtide (3.65 and 3.72 mg) were used to prepare stock solutions. After accurate dissolution in 10 mL ACN/water (1/1, v/v) + 0.1% FA using volumetric flasks, resulting solutions were diluted 10-fold with the same solvent into polypropylene vessels. Spike solutions (polypropylene vessels) for preparation of calibration samples were generated from the diluted stock at concentrations of 0.4, 1.2, 4, 12, 40, 120, 400, 1200 and 4000 ng/mL in ACN/water (1/1, v/v) + 0.1% FA, which corresponds to sample concentrations of 0.1, 0.3, 1, 3, 10, 30, 100, 300, and 1000 ng/mL (0.1 to 100 ng/mL for the low concentration assay; 1 to 1000 ng/mL for the high concentration assay). Spike solutions for preparation of QC samples were prepared accordingly at 0.4, 1.2, 4, 12, 150, 300, 1500, 3000 and 20,000 ng/mL, which corresponds to sample concentrations of 0.1, 0.3, 1, 3, 37.5, 75, 375, 750, and 5000 ng/mL (LLOQ 0.1, QC A, LLOQ 1.0, QC B, QC C, QC D, QC E, QC F, and dilution QC, respectively). LLOQ 0.1, QC A, QC C, and QC D were applied to the low concentration assay; LLOQ 1.0, QC B, QC E, QC F, and the dilution QC were applied to the high concentration assay. The IS spike solutions were similarly prepared at 40 and 400 ng/mL (corresponding sample concentration of 10 and 100 ng/mL). For the purpose of plasma stability investigations, QC spike solutions for minimally diluted plasma samples (2% solvent) were prepared at 150, 18,750, and 37,500 ng/mL (corresponding sample concentrations of 3, 375, and 750 ng/mL). Solutions were kept at 4 °C.

### Plasma sample preparation

Calibration and QC plasma samples were generated in 2-mL reaction tubes from 100 μL of blank pooled plasma by addition of 25 μL of IS and 25 μL of the respective calibration or QC spike solution. Dilution QC samples were prepared by addition of 25 μL of dilution QC spike solution to 75 μL blank plasma and the subsequent dilution of 10 μL of this dilution QC sample with 90 μL blank plasma followed by the addition of 25 μL of IS solution and 25 μL of ACN/water (1/1 + 0.1% FA, v/v) for volume compensation. Study plasma samples (100 μL) were also spiked with 25 μL of IS solution and 25 μL of ACN/water (1/1 + 0.1% FA, v/v) for volume compensation. Samples for stability evaluations were prepared by adding 20 μL of the respective stability QC spike solution to 980 μL of plasma without addition of IS, which was spiked after storage.

Plasma samples were depleted of proteins by their addition to 300 μL of ACN + 0.1% FA present in wells of Impact® protein precipitation plates (Phenomenex, Torrance, CA, USA), and subsequent agitation for 2 min. Extracts were generated by transfer of the protein precipitated samples through the filter of the plate to a 96-well collection plate (800 μL; Waters) using positive pressure (3–6 psi; positive pressure unit, Waters).

For the high concentration assay, extracts were diluted with 200 μL of water + 0.1% FA, plates sealed, and sample extracts vortex-mixed.

For the low concentration assay, the extracts were concentrated to approximately 50 μL with heated nitrogen (35 °C) applied for 25 min at 3 psi with a blowdown evaporator (Ultravap®, Porvair Sciences, Wrexham, Wales, UK). Afterwards, 100 μL of MeOH + 0.1% FA was added to each well, plates sealed, and sample extracts vortex-mixed.

### Instrumental analysis parameters

Measurements for the high concentration assay were performed on an I-class UPLC® system (Waters) connected to a Xevo TQ-S (Waters) triple quadrupole mass spectrometer. For the low concentration assay, quantification was done on an Acquity classic UPLC® system (Waters) coupled to a Xevo TQ-XS (Waters) triple quadrupole mass spectrometer. Both MS systems were equipped with a heated ESI source (Z-spray). Capillary voltage was manually optimized. The remaining mass spectrometric parameters were tuned with the integrated IntelliStart procedures of the MassLynx system software (V4.2 and V4.1; Waters). Argon was used for CID in positive ion mode SRM measurements. A summary of the mass spectrometric characteristics is shown in the Supplementary Information (ESM) Table S1.

High-resolution MS (HRMS) analyses were performed on a G2-XS QToF mass spectrometer (Waters) with a Z-Spray ESI source using the integrated direct infusion system and a 1000 ng/mL solution of bulevirtide in ACN/water (1/1, v/v) + 0.1% FA.

Eluents for chromatographic separation were as follows: water including 5% of ACN and 0.1% of FA (eluent A) and ACN including 0.1% FA (eluent B). Separation was achieved using an Acquity Premier BEH C18 peptide column (300 Å, 1.7 μm, 2.1 × 50 mm; Waters) with a flow rate of 0.5 mL/min, which was maintained at 60 °C. A sample injection volume of 20 μL was used. A gradient from 30% B to 85% B in 1.5 min, which started 0.5 min of maintaining initial conditions, was found optimal. Following this separation gradient, the column was flushed by changing the composition to 98% B within 0.1 min and maintaining this condition for additional 0.4 min before returning to initial condition in 0.5 min, which were maintained for approximately 1 min during preparation of the following injection by the Sample Manager. This resulted in a total sample cycle time of 4 min.

### Validation of the analytical methods

Validation parameters were defined according to the pertinent recommendations of the FDA and EMA [17, 18]. Validation was performed in regard to accuracy and precision within-run and run-to-run in three validation runs. Each run contained blank plasma, one zero, and the seven non-zero calibration concentrations, each in double determination, as well as six determinations of four QC concentrations (LLOQ, low QC at three-times the LLOQ, mid QC at 37.5% of the ULOQ, and high QC at 75% of the ULOQ). Accuracy was calculated from the mean determined concentration of QC samples as percent of the nominal value. The respective acceptance limits are within ±15% of the nominal value (100%) with the exception of the LLOQ, which must lie within ±20%. Precision was evaluated from the standard deviation of measured QC samples as percent of the mean determined concentration. Required limits are ≤15% in general and ≤ 20% at the LLOQ.

Selectivity was evaluated in six different individual lots of blank plasma for absence of interfering peaks at the analyte retention time (peak area ≤ 20% of LLOQ and ≤ 5% of IS). Extraction recovery rates from plasma were assessed from low to high QC samples in three-fold determination as ratio of response in regard to blank plasma spiked after extraction (representing 100% analyte amount in identical matrix) expressed in percent. Matrix effects were determined also in three-fold determination for low to high QC concentration via the comparison of the response of blank plasma samples spiked after extraction with the respective response of matrix-free extract solvent spiked with the identical amount. [19]

Reliability of quantification was assessed with clinical study samples in a cross-validation study and incurred sample reanalysis (ISR) investigations applying the recommended limits that at least 67% of reanalyzed samples must show a deviation of <20% of the mean value of original and reanalysis.

Stability of bulevirtide was assessed for plasma samples stored at −20 °C for 14 days, as well as in three freeze-and-thaw cycles using low to high QC samples. Stability of the extracts in the autosampler was evaluated by a repeated analysis of QC samples after remaining in the autosampler at 15 °C for at least 24 h with freshly processed calibration samples. Extract stability was evaluated through quantification using peak areas (external analysis) to avoid compensation of possible degradation by the IS, which possesses identical physicochemical characteristics. Furthermore, stock solution stability was established by quantification of stored QC spike solutions with calibration solutions prepared from an independent fresh weighing of bulevirtide.

### Calculations and statistical methods

Calibration curves were calculated with weighted linear regressions (1/x^2^) from the response (peak area ratios of the analyte and IS) of calibration samples with the TargetLynx software (V4.2 or V4.1; Waters). Pharmacokinetic parameters were calculated using Thermo Kinetica version 5.0 (Thermo Fisher Scientific, Waltham, MA, USA). Remaining calculations were performed with Microsoft Office Excel 2010 (Mountain View, CA, USA).

## Results and discussion

### Mass spectrometric characteristics

**Fig. 1:**
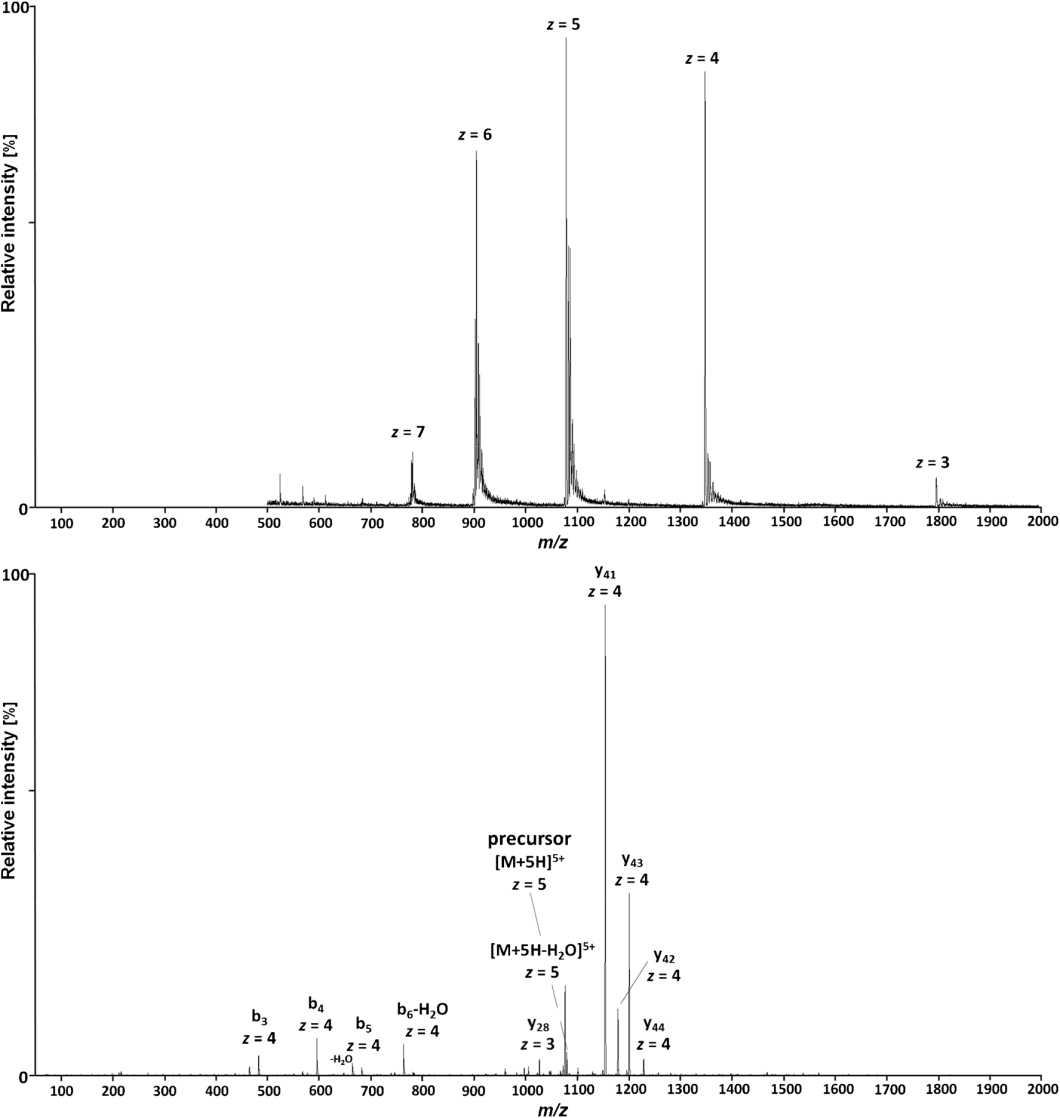
Positive precursor of bulevirtide (on top) and product ion spectrum of the [M+5H]^5+^ signal at *m*/*z* 1080.8 of bulevirtide using collision-induced dissociation at 22 V (below)

Positive electrospray ionization of bulevirtide (C_248_H_355_N_65_O_72_, 5398.9 g/mol) yielded a number of multiply charged species with the [M+5H]^5+^ signal at *m*/*z* 1080.8 as most intense precursor ion (*m*/*z* 1086.6 for the IS). However, the abundance of the [M+4H]^4+^ signal at *m*/*z* 1350.7 was not far behind. The multiple *z* = 6 species showed higher abundance for the alkali metal salts, as were the substantially less intense *z* = 7 signals. The last remaining observed charge state (*z* = 3) was represented by the [M+3H]^3+^ ion, however at low intensity. The most abundant protonated precursor ions at the charge state of 4 and 5 were investigated for their CID characteristics (the *z* = 6 ions showed substantially lower product ion intensities than the other two abundant ions as expected for alkali metal adducts). Bulevirtide generates only few abundant, large (and therefore selective) product ions in CID, which results in only a moderate intensity loss for SRM, considering the possible number of non-selective peptide bond dissociations. For the most intense precursor ions at *z* = 4 and *z* = 5, the y_41_ product ion was the most abundant showing the highest maximal intensity with optimized collision energy, however differently charged at *z* = 3 (*m*/*z* 1554.4) and *z* = 4 (*m*/*z* 1155.2), respectively. In comparison, CID of the [M+5H]^5+^ signal yielded substantially higher intensity for the y_41_ product ion (*z* = 4) compared to the [M+4H]^4+^ ion. Figure 1 shows the MS spectrum of bulevirtide and the product spectrum of the [M+5H]^5+^ precursor ion with the most intense fragments assigned (the assignment was supported by HRMS). Bulevirtide produces primarily few large fragments in CID. The fragment pattern is identical for the *z* = 5 and *z* = 4 precursor ion with only the charge state of the product ions being one greater for the [M+5H]^5+^ precursor. The abundant fragments are generated primarily from the respective b and y-fragments of four adjacent peptide bond dissociations located close to the N-terminus of bulevirtide. In addition, an abundant product ion is generated by water loss. These include the b/y-pairs: b_3_ (and b_3_-H_2_O)/y_44_, b_4_/y_43_, b_5_ (and b_5_-H_2_O)/y_42_, b_6_-H_2_O/y_41_, and the respective [M-H_2_O+nH]^n+^ ion resulting from water loss of the precursor. The observed preference of these peptide bond dissociations may indicate a favorable protonation by mobile protons in the respective N-terminal region of bulevirtide.

The high intensity of the y_41_ product ion is likely caused by the favored dissociation of peptide bonds at the N-terminal side of prolines (“proline effect”; [20–27]). The fact that from the remaining six proline residues the only further detected fragment, the y_28_ product ion, is a magnitude less intense than the y_41_ fragment, again may indicate that the amide bonds in the N-terminal region (3 to 6) of bulevirtide exhibits favorable basicity for protonation by mobile protons. As a consequence of the few preferential peptide bond dissociations in CID, and the resulting abundant large fragments, which foster high selectivity of the monitored mass transition, highly sensitive quantification with MS/MS measurements is possible for bulevirtide.

Because of low background and absence of interfering signals for the most abundant and large fragment at *m*/*z* 1080.8, the corresponding mass transition *m*/*z* 1080.8 → 1155.2 was chosen for quantification using SRM. To assure that the IS accounts for variations in mass spectrometric detection, the corresponding mass transition of *m*/z 1086.6 → 1162.4 was monitored for the IS.

### Chromatographic characteristics

**Fig. 2:**
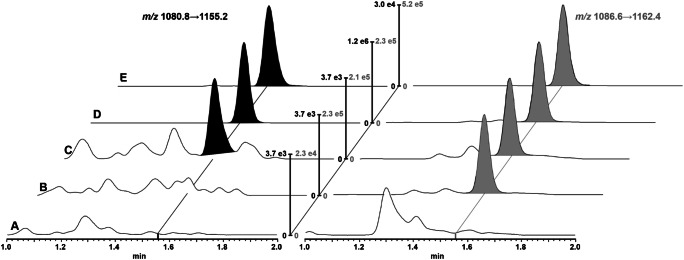
UPLC-MS/MS ion chromatograms of processed plasma samples. The bulevirtide transition is shown on the left (black) and the IS transition is shown on the right (gray). A, blank plasma sample; B, plasma sample with added IS; C, plasma sample at LLOQ concentration (0.1 ng/mL); D, plasma sample at QC C concentration (37.5 ng/mL); and E, trough plasma sample of volunteer #3 at steady-state determined in cross-validation (quantified bulevirtide concentration 0.722 ng/mL). Normalization was performed to the highest signal, except for the bulevirtide transition of A and B, which were adjusted to the LLOQ, and the IS transition of A, which was adjusted to a tenth of the blank sample with added IS (B)

Because of its considerable size, bulevirtide was chromatographically separated via UPLC on a column with a large pore width of 300 Å maintained at 60 °C to foster optimal mass transfer kinetics. Due to its size and lipophilicity, which is further increased by the N-terminal fatty acid modification, bulevirtide exhibits a substantial tendency for non-specific absorption. Therefore, to avoid absorption loss and to reduce carry-over, we used a column with very little blank metal surface, a Waters Premier BEH C18 column, which features a cover of the inner surface of the column housing with the BEH C18 material. A rapid gradient from 30 to 85% ACN eluent within 1.5 min was found optimal in regard to separation of potential interfering signals and sample throughput. Figure 2 shows typical ion chromatograms obtained during bulevirtide assay validation and clinical study sample quantification. The observed carry-over in both assays was 0.06% and therefore three times higher than required for absence of influence on sample analysis, because its extent corresponds to approximately 60% of LLOQ peak area. Nevertheless, its dimension is very low and only samples very close to the LLOQ following samples very close to the ULOQ may be influenced. Because no measures were able to further reduce the carry-over, we decided to allow for influenced clinical samples to be reanalyzed. However, during the course of clinical study measurements, no sample constellations with the need of reanalysis were observed.

### Extraction by protein precipitation

For the high concentration assay, we established a fast protein precipitation procedure simply consisting of protein precipitation in 96-well format with subsequent dilution with water. This resulted in a fast sample processing method beneficial for high sample throughput. In the course of method development for the low concentration assay, we tested three different solid phase extraction materials in μ-elution format with and without prior protein precipitation, and compared it in-run with sole protein precipitation followed by concentration of the extracts by evaporation. All tested SPE materials (cationic and anionic exchange as mixed mode reverse phase material, as well as pure reverse phase material; MCX, MAX, and HLB; Oasis®, Waters) showed very little recovery, which we account primarily to (too) high retention of bulevirtide. Peak area comparisons revealed that protein precipitation followed by solvent evaporation was superior compared to all tested SPE materials. As a consequence, bulevirtide extraction was performed using protein depletion by precipitation with ACN, which additionally shows the benefit of being well-manageable and efficient when performed in 96-well format precipitation plates. The recovery in both assays of all QC concentrations and both IS concentrations ranged between 49.3 and 53.8% and therefore is consistent across the whole concentration range ensuring reliable quantification. Table S2 in the ESM shows the detailed recovery data obtained during validation. Moderate recovery is not uncommon for large and lipophilic peptides due to incomplete extraction because of co-precipitation during protein depletion [14].

A common characteristic of protein-precipitated extracts in peptide bioanalysis is the occurrence of a considerable matrix effect in ESI [6], which is generally attributed to ion suppression by remaining phospholipids and the occurrence of sodium adducts. Because of the higher amount of matrix components in the concentrated extracts, the observed matrix effects are more pronounced for the low concentration assay. The observed absolute matrix effects in the six individual plasma lots in the low concentration assay with values ranging from 36.3 to 57.1% (−73.7 to −42.9%) are approximately double those of the high concentration assay, which showed matrix effects between 72.3 and 102.2% (−27.7 to 2.2%). The IS reliably balanced the analyte matrix effects as reflected by the IS-normalized matrix effects in both assays, which ranged from 92.0 to 109.5% well fulfilling the regulatory requirements. A detailed summary of the matrix effect data is given in ESM Tables S3 and S4.

In lipemic and hemolytic QC plasma samples, peak areas were substantially decreased and the IS-normalized recovery and matrix effects were in part above the recommended limits (see ESM Tables S3 and S4). Therefore, such sample determinations may lack reliability and need to be excluded from sample analysis.

### Validation results

Bulevirtide quantification by UPLC-MS/MS after extraction with protein precipitation completely complied with the pertinent guidelines of FDA and EMA [17, 18]. No interferences were observed in six individual lots of blank plasma with both sample processing methods, demonstrating the selectivity of the assays. Linearity of the calibration curves was proven by correlation coefficients (*r*^2^) of all calibration curves being >0.98 (weighted (1/x^2^) linear regression; see ESM Table S5). The inter-run and run-to-run accuracies of both assays were between 88.8 and 104.6% (86.0 and 104.7% at LLOQ) with corresponding precision ≤10.2% (≤ 16.1% at LLOQ). These results are well in compliance with the recommended requirements. The obtained quality control results are summarized in Tables 1 and 2. In addition, the accurate determination of minimally diluted QC plasma samples with calibration plasma samples spiked with 25 μL of QC solutions demonstrated that our sample preparation methodology is feasible for study plasma sample measurements (see ESM Table S6).

**Table 1:** Accuracy data of the plasma validation

QC level	Nominal concentration (ng/mL)	Accuracy (%)			
		Within-batch			Batch-to-batch
		Batch #1	Batch #2	Batch #3	
LLOQ low	0.10	96.5	97.7	104.7	99.6
QC A	0.30	94.8	95.5	98.5	96.1
LLOQ high	1.0	86.0	98.3	94.3	92.3
QC B	3.0	94.5	94.1	92.5	93.8
QC C	37.5	103.8	101.1	102.4	102.4
QC D	75	104.6	97.6	102.7	101.6
QC E	375	92.2	93.3	90.4	92.1
QC F	750	88.8	88.9	91.7	89.7

*LLOQ* lower limit of quantification, *QC* quality control. *N* = 6 replicates

**Table 2:** Precision data of the plasma validation

QC level	Nominal concentration (ng/mL)	Precision (%)			
		Within-batch			Batch-to-batch
		Batch #1	Batch #2	Batch #3	
LLOQ low	0.10	4.4	9.2	16.1	11.5
QC A	0.30	5.4	4.9	10.2	7.1
LLOQ high	1.0	3.6	4.4	5.1	5.6
QC B	3.0	4.0	5.8	6.5	5.1
QC C	37.5	1.3	4.7	1.9	3.0
QC D	75	1.9	4.6	3.8	4.5
QC E	375	1.0	1.5	4.9	2.9
QC F	750	2.0	2.0	2.0	2.3

*LLOQ* lower limit of quantification, *QC* quality control. *N* = 6 replicates

Due to an accuracy of 97.9% and corresponding precision of 2.9%, the dilution integrity for 10-fold dilution with blank plasma was established for the high concentration assay, extending the quantification range to a maximum of 10,000 ng/mL. Due to the ease of sample processing for the high concentration assay, which simplifies clinical study sample throughput, we did not establish dilution integrity for the low concentration assay. However, clinical samples were used to cross-validate both assays to establish their interchangeable use (eliminating the need for dilution of the low concentration assay). The cross-validation was in full compliance with the pertinent recommendations because 81% of samples showed a deviation of <20% from the mean of both quantifications (see ESM Table S7). Furthermore, an ISR was performed for both assays also using the clinical study samples. Due to sample volume restrictions, different numbers of samples were utilized in the two ISR measurements. In the high concentration assay, all 42 reanalyzed samples showed a deviation of <20% from the mean (see ESM Table S8), and in the low concentration assay of 27 reanalyzed samples 78% had a deviation <20% from the mean (see ESM Table S9). Again, these results were well in compliance with the guidelines of the FDA and EMA [17, 18], which clearly underlines the reliability of the bulevirtide bioanalysis.

### Stability

In support of validation, the stability of bulevirtide was established in plasma during freeze-and-thaw and for storage at −20 °C. To guarantee reliable quantification, the stability of bulevirtide in the prepared stock solutions and in the extracts was also established for the expected course of clinical sample analysis and analytical run time. Bulevirtide was found stable during three freeze-and-thaw cycles with accuracies ranging from 86.5 to 107.7% (precision ≤3.0%). Because thawed samples were kept at room temperature for at least 2 h, stability under these conditions for the required time span of sample preparation was also confirmed. Additionally, bulevirtide’s integrity in plasma during storage was demonstrated for at least 14 days at −20 °C with determined accuracies of 107.2 to 108.8% (precision ≤1.0%). Stability of bulevirtide in the extracts was confirmed for at least 24 h by determined accuracies ranging from 88.8 to 111.8% (precision ≤5.7%) for QC samples stored in the autosamplers of the respective UPLC systems. Furthermore, bulevirtide was found stable for at least 7 weeks in solution at 4 °C, which was demonstrated with quantification of freshly prepared QC solutions from a fresh weighing with stored calibration samples resulting in accuracies of 101.6 to 110.2% (precision ≤5.8%). Details of the determined stability data are shown in the ESM Tables S10 to S13.

### Bulevirtide plasma concentrations after single dose and at steady-state

**Fig. 3:**
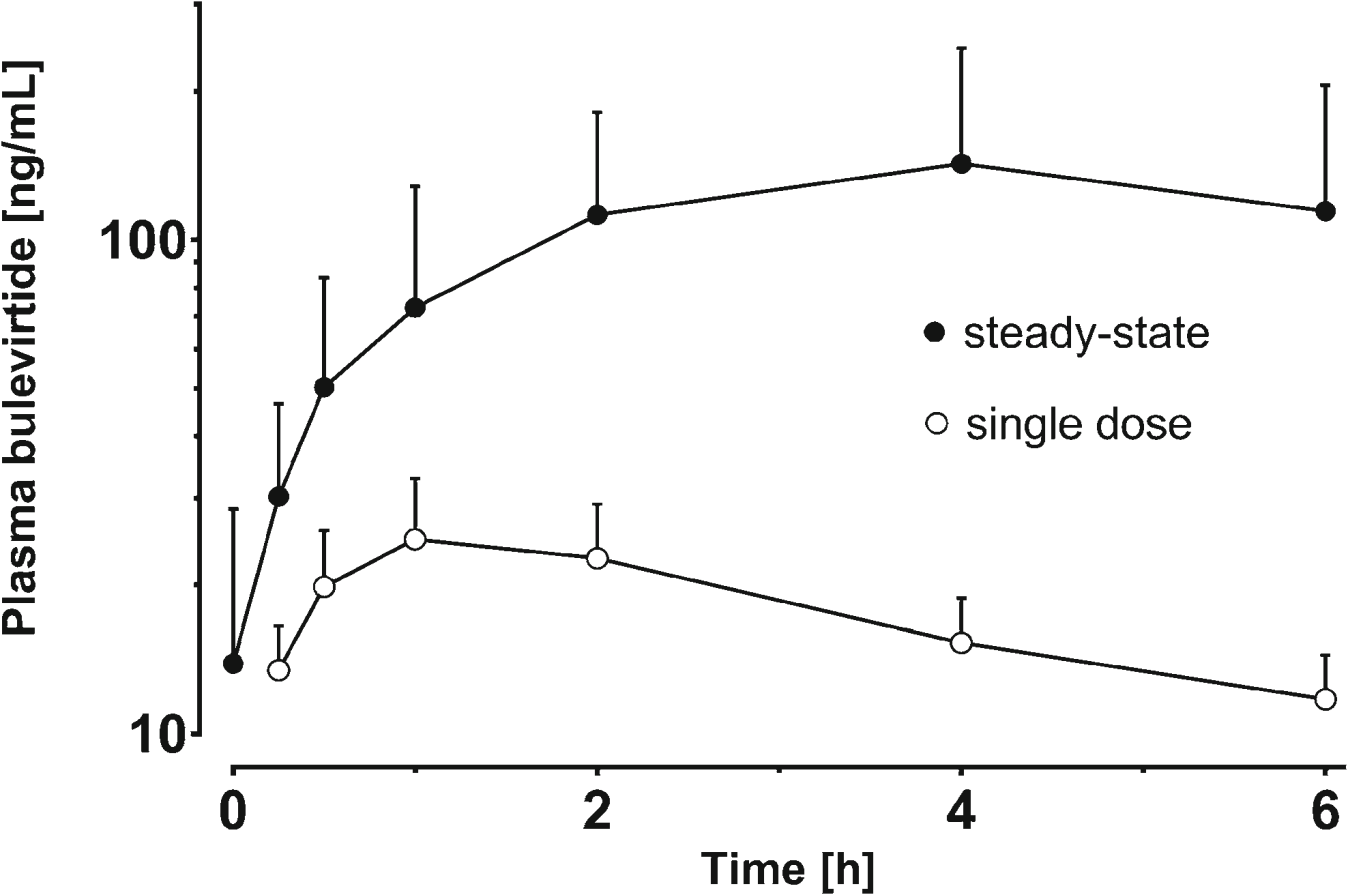
Plasma concentration-time profiles of bulevirtide in 14 volunteers after single-dose administration and at steady-state. Concentrations are presented as geometric mean with 95% CI

From the enrolled 14 volunteers, 13 completed the trial and were included in the pharmacokinetic evaluation. Single-dose and steady-state blood samples were determined with the high concentration assay. Figure 3 shows the obtained concentration-time profiles of plasma bulevirtide after single dose and at steady-state. Part of these samples (single-dose samplings of 6 volunteers) were also measured with the low concentration assay for the purpose of cross-validation. Additionally, samples were used for two independent ISR for both assays. These reanalysis investigations are crucial in assessing the validity of bulevirtide bioanalysis because they demonstrate the reliability of real-world sample determinations. As discussed before, ISR and cross-validation were well within the recommended limits underpinning the validity of the measurements and the established bioanalysis.

Because of the limited sampling time, pharmacokinetic parameters could not be determined in detail. However, the assessed parameters, which are shown in Table 3, were reasonably concordant with previously obtained results [3, 28]. Peak concentrations were reached substantially later at steady-state compared to the single dose, while the obtained *C*_max_ and the area under the concentration-time curve from 0 to 6 h were significantly higher (4.7-fold and 5.4-fold, respectively). Compared to previous data [28], this is an increased elevation; however, in the here-presented exploratory trial, steady-state plasma concentrations were assessed considerably later after dosing initiation (85 vs. 5 days).

**Table 3:** Determined pharmacokinetic parameters of 5 mg subcutaneous bulevirtide

Pharmacokinetic parameter^a^	Single dose	Steady-state
AUC_0-6_ (ng/mL×h)	97.0 (78.3, 120)	526 (370, 747)
*C*_max_ (ng/mL)	24.8 (19.3, 31.8)	117 (79.6, 173)
*T*_max_ (h)^b^	1.29 (0.50, 2.00)	3.77 (1.00, 4.00)

^a^Geometric means with 95% CI. ^b^Median and range

*AUC*_*0-6*_, area under the concentration-time curve from 0 to 6 h; *C*_*max*_, maximal plasma concentration; *T*_*max*_, time to reach maximal plasma concentration

## Conclusion

We developed a well-manageable and highly sensitive bioanalysis for the very large lipopeptide bulevirtide relying on intact peptide determination with UPLC-MS/MS. The established concentration range of quantification of two established assays spans five orders of magnitude with an LLOQ of 0.1 ng/mL (19 pM). Because of the favored CID of peptide bonds in the N-terminal region and especially of that adjacent to proline, bulevirtide generates selective, large, and abundant fragments suitable for highly sensitive SRM. Both assays were fully validated according to the regulatory requirements and additionally cross-validated with clinical samples of an exploratory phase I trial demonstrating their interchangeable use. Successful incurred sample reanalyses of the clinical samples further supported the validity of real-world sample bulevirtide quantification, clearly demonstrating the assays’ reliability for regulatory bioanalysis.

## Supplementary information

ESM 1 (PDF 425 kb)

## Data Availability

Data is available within the article and the ESM.

## References

[1] Ni Y, Lempp FA, Mehrle S, Nkongolo S, Kaufman C, Falth M (2014). Hepatitis B and D viruses exploit sodium taurocholate co-transporting polypeptide for species-specific entry into hepatocytes. Gastroenterology.

[2] Yurdaydin C, Abbas Z, Buti M, Cornberg M, Esteban R, Etzion O, Gane EJ, Gish RG, Glenn JS, Hamid S, Heller T, Koh C, Lampertico P, Lurie Y, Manns M, Parana R, Rizzetto M, Urban S, Wedemeyer H (2019). Treating chronic hepatitis delta: the need for surrogate markers of treatment efficacy. J Hepatol.

[3] Bogomolov P, Alexandrov A, Voronkova N, Macievich M, Kokina K, Petrachenkova M, Lehr T, Lempp FA, Wedemeyer H, Haag M, Schwab M, Haefeli WE, Blank A, Urban S (2016). Treatment of chronic hepatitis D with the entry inhibitor myrcludex B: first results of a phase Ib/IIa study. J Hepatol.

[4] Blank A, Markert C, Hohmann N, Carls A, Mikus G, Lehr T, Alexandrov A, Haag M, Schwab M, Urban S, Haefeli WE (2016). First-in-human application of the first-in-class hepatitis B and hepatitis D virus entry inhibitor myrcludex B. J Hepatol.

[5] Rauh M (2012). LC-MS/MS for protein and peptide quantification in clinical chemistry. J Chromatogr B Anal Technol Biomed Life Sci.

[6] van den Broek I, Sparidans RW, Schellens JH, Beijnen JH (2008). Quantitative bioanalysis of peptides by liquid chromatography coupled to (tandem) mass spectrometry. J Chromatogr B Anal Technol Biomed Life Sci.

[7] Sauter M, Uhl P, Burhenne J, Haefeli WE (2021). Application of triple quadrupole tandem mass spectrometry to the bioanalysis of collision-induced dissociation-resistant cyclic peptides − ultra-sensitive quantification of the somatostatin-analog pasireotide utilizing UHPLC-MS/MS. J Pharm Biomed Anal.

[8] Sauter M, Uhl P, Burhenne J, Haefeli WE (2020). Post-extraction disulfide bond cleavage for MS/MS quantification of collision-induced dissociation-resistant cystine-cyclized peptides and its application to the ultra-sensitive UPLC-MS/MS bioanalysis of octreotide in plasma. Anal Chim Acta.

[9] Sauter M, Uhl P, Burhenne J, Haefeli WE (2020). Ultra-sensitive quantification of the therapeutic cyclic peptide bremelanotide utilizing UHPLC-MS/MS for evaluation of its oral plasma pharmacokinetics. J Pharm Biomed Anal.

[10] Fu Y, Xia YQ, Flarakos J, Tse FL, Miller JD, Jones EB, Li W (2016). Differential mobility spectrometry coupled with multiple ion monitoring in regulated LC-MS/MS bioanalysis of a therapeutic cyclic peptide in human plasma. Anal Chem.

[11] Klaassen T, Szwandt S, Kapron JT, Roemer A (2009). Validated quantitation method for a peptide in rat serum using liquid chromatography/high-field asymmetric waveform ion mobility spectrometry. Rapid Commun Mass Spectrom.

[12] Xia YQ, Wu ST, Jemal M (2008). LC-FAIMS-MS/MS for quantification of a peptide in plasma and evaluation of FAIMS global selectivity from plasma components. Anal Chem.

[13] Levin DS, Miller RA, Nazarov EG, Vouros P (2006). Rapid separation and quantitative analysis of peptides using a new nanoelectrospray- differential mobility spectrometer-mass spectrometer system. Anal Chem.

[14] Meng X, Xu H, Zhang Z, Fawcett JP, Li J, Yang Y, Gu J (2017). Differential mobility spectrometry tandem mass spectrometry with multiple ion monitoring for the bioanalysis of liraglutide. Anal Bioanal Chem.

[15] Sauter M, Uhl P, Majewsky M, Fresnais M, Haefeli WE, Burhenne J (2019). An ultra-sensitive UPLC-MS/MS assay for the quantification of the therapeutic peptide liraglutide in plasma to assess the oral and nasal bioavailability in beagle dogs. Bioanalysis.

[16] Sauter M, Uhl P, Burhenne J, Haefeli WE (2020). Ultra-sensitive bioanalysis of the therapeutic peptide exenatide for accurate pharmacokinetic analyses at effective plasma concentrations utilizing UPLC-MS/MS. J Pharm Anal.

[17] US Department of Health and Human Services, Food and Drug Administration, Guidance for industry, bioanalytical method validation. 2018. http://www.fda.gov/downloads/Drugs/GuidanceComplianceRegulatoryInformation/Guidances/ucm070107.pdf. Accessed Jan 2021.

[18] Committee for Medicinal Products for Human Use, European Medicines Agency, Guideline on validation of bioanalytical methods EMEA/CHMP/EWP/192217/2009. 2009. http://www.ema.europa.eu/docs/en_GB/document_library/Scientific_guideline/2011/08/WC500109686.pdf. Accessed Jan 2021.

[19] Matuszewski BK, Constanzer ML, Chavez-Eng CM (2003). Strategies for the assessment of matrix effect in quantitative bioanalytical methods based on HPLC-MS/MS. Anal Chem.

[20] Loo JA, Edmonds CG, Smith RD (1993). Tandem mass spectrometry of very large molecules. 2. Dissociation of multiply charged proline-containing proteins from electrospray ionization. Anal Chem.

[21] Schwartz BL, Bursey MM (1992). Some proline substituent effects in the tandem mass spectrum of protonated pentaalanine. Biol Mass Spectrom.

[22] Vaisar T, Urban J (1996). Probing the proline effect in CID of protonated peptides. J Mass Spectrom.

[23] Harrison AG, Young AB (2005). Fragmentation reactions of deprotonated peptides containing proline. The proline effect. J Mass Spectrom.

[24] Grewal RN, El Aribi EH, Harrison AG, Siu KWM, Hopkinson AC (2004). Fragmentation of protonated tripeptides: the proline effect revisited. J Phys Chem B.

[25] Huang Y, Triscari JM, Tseng GC, Pasa-Tolic L, Lipton MS, Smith RD, Wysocki VH (2005). Statistical characterization of the charge state and residue dependence of low-energy CID peptide dissociation patterns. Anal Chem.

[26] Unnithan AG, Myer MJ, Veale CJ, Danell AS (2007). MS/MS of protonated polyproline peptides: the influence of N-terminal protonation on dissociation. J Am Soc Mass Spectrom.

[27] Bleiholder C, Suhai S, Harrison AG, Paizs B (2011). Towards understanding the tandem mass spectra of protonated oligopeptides. 2. The proline effect in collision-induced dissociation of protonated Ala-Ala-Xxx-Pro-Ala (Xxx = Ala, Ser, Leu, Val, Phe, and Trp). J Am Soc Mass Spectrom.

[28] Blank A, Eidam A, Haag M, Hohmann N, Burhenne J, Schwab M, van de Graaf S, Meyer MR, Maurer HH, Meier K, Weiss J, Bruckner T, Alexandrov A, Urban S, Mikus G, Haefeli WE (2018). The NTCP-inhibitor myrcludex B: effects on bile acid disposition and tenofovir pharmacokinetics. Clin Pharmacol Ther.

