# Supplementary material for: Intact plasma quantification of the large therapeutic lipopeptide bulevirtide
Source: Anal Bioanal Chem. 2021 May 20;413(22):5645–54. doi: 10.1007/s00216-021-03384-7 (PMC8410713; doi:10.1007/s00216-021-03384-7)
Supplement: Supplementary file 1 — (PDF 425 kb) [file 216_2021_3384_MOESM1_ESM.pdf]

Intact plasma quantification of the large therapeutic lipopeptide bulevirtide

Max Sauter[1,2], Antje Blank[1,2], Felicitas Stoll[1,2], Natalie Lutz[1,2], Walter E. Haefeli[1,2], Jürgen Burhenne[1,2,*]

[1] Department of Clinical Pharmacology and Pharmacoepidemiology, Heidelberg University Hospital, Im Neuenheimer Feld 410, 69120 Heidelberg, Germany

[2] German Center for Infection Research (DZIF), Heidelberg Partner Site, Im Neuenheimer Feld 410, 69120 Heidelberg, Germany

Supplementary Information

Table S1: Optimized MS/MS parameters for the detection of bulevirtide using heated ESI and SRM in the positive ion mode.

Parameter	High concentration assay	Low concentration assay
**MS system**	**Xevo TQ-S**	**Xevo TQ-XS**
Capillary voltage [kV]	0.8	1.8
Cone voltage [V]	12	20
Source temperature [°C]	150	
Desolvation temperature [°C]	600	
Cone gas (N2) flow [L/h]	150	
Desolvation gas (N2) flow [L/h]	1000	
BLT mass transition SRM [m/z]	1080.8 → 1155.2	
IS mass transition SRM [m/z]	1086.6 → 1162.4	
Collision gas (Ar) flow [mL/min]	0.15	
Collision energy [V]	17	22

ESI: Electrospray ionization; IS: internal standard; SRM: selected reaction monitoring.

Table S2: Recovery data of the plasma validation.

QC level	Recovery [%]			SIL-IS normalized recovery [%]		
	Plasma lot #					
	Pool	Hem	Lip	Pool	Hem	Lip
QC A	51.3	24.3	57.8	102	126	101
QC B	53.0	13.4	14.8	107	98	119
QC C	51.5	19.3	53.2	95	95	95
QC D	53.8	25.8	58.1	95	100	98
QC E	52.2	12.5	15.8	111	135	108
QC F	52.6	12.9	14.1	115	168	108
Low IS	53.6			95	100	98
High IS	49.3					

Hem: hemolytic plasma; IS: internal standard; Lip: lipemic plasma; Pool: pooled plasma.

N = 3 replicates at each QC concentration.

Table S3: Matrix effect data.

QC level	Matrix effect [%]								
	Plasma lot #								
	#12	#13	#14	#16	#17	#18	Pool	Hem	Lip
QC A	44.1	54.2	49.5	44.0	55.4	57.1	55.4	56.5	18.6
QC B	75.6	77.0	72.8	72.3	80.3	79.9	82.3	80.3	50.5
QC C	36.7	45.0	38.8	43.2	48.9	49.6	44.2	48.4	17.1
QC D	36.3	43.1	37.4	41.6	46.2	45.6	43.6	41.6	15.9
QC E	96.3	102.2	97.2	92.0	98.2	96.0	91.3	96.4	56.3
QC F	90.0	94.5	93.5	88.2	94.0	90.1	86.8	90.9	55.8

Hem: hemolytic plasma; Lip: lipemic plasma; Pool: pooled plasma.

N = 3 replicates at each QC concentration.

Table S4: IS-normalized matrix effect data.

QC level	IS normalized matrix effect [%]								
	Plasma lot #								
	#12	#13	#14	#16	#17	#18	Pool	Hem	Lip
QC A	107.3	107.3	108.5	98.8	107.3	97.6	108.5	106.1	107.3
QC B	109.5	107.4	105.4	106.8	106.8	106.1	108.8	110.8	113.5
QC C	94.6	97.1	92.0	97.6	99.3	97.3	97.4	103.9	103.7
QC D	96.8	95.7	93.2	94.5	96.2	95.8	98.6	100.2	100.7
QC E	93.6	94.5	94.6	95.7	96.0	100.7	101.0	98.6	101.7
QC F	92.3	94.5	95.3	96.8	98.0	99.5	101.6	99.3	100.8

Hem: hemolytic plasma; IS: internal standard; Lip: lipemic plasma; Pool: pooled plasma.

N = 3 replicates at each QC concentration.

Table S5: Calibration curves of validation batches calculated with linear regression and $1/x^2$ weighting.

Validation batch	R^2	Equations
#1 high	0.9985	y = 0.01884 x + 0.00079
#2 high	0.9985	y = 0.01945 x + 0.00094
#3 high	0.9944	y = 0.01856 x + 0.00067
#1 low	0.9962	y = 0.0915 x + 0.0012
#2 low	0.9946	y = 0.1013 x + 0.0005
#3 low	0.9894	y = 0.0961 x + 0.0018

Table S6: Accuracy and precision data for the determination of minimally diluted plasma QC with spiked calibration samples.

QC level	Accuracy [%]	Precision [% CV]
QC B	107.8	3.5
QC E	104.4	4.1
QC F	108.2	1.5

N = 3 replicates at each QC concentration.

Table S7: Cross-validation of low and high concentration assays.

Volunteer #	Sampling time [h]	Original analysis [ng/mL]	Reanalysis [ng/mL]	Deviation from mean [%]
1	0.25	6.30	7.58	18.5
1	0.5	9.12	11.02	18.9
1	1	14.7	16.89	13.8
1	2	18.9	19.87	5.0
1	4	11.4	13.63	17.8
1	6	8.20	10.0	20.1
1	SS-0	46.2	52.5	12.8
2	0.25	11.0	13.2	17.9
2	0.5	13.8	16.6	18.2
2	1	15.1	15.8	4.3
2	2	17.4	19.7	12.2
2	4	13.6	13.0	4.8
2	6	9.54	9.9	4.0
2	SS-0	1.56	1.73	10.1
3	0.25	20.7	18.9	9.0
3	0.5	25.7	27.8	7.9
3	1	24.4	23.1	5.3
3	2	20.0	30.6	42.0
3	4	19.1	18.7	2.1
3	6	17.4	17.1	1.7
3	SS-0	0.856	0.722	17.0
4	0.25	11.0	14.4	26.5
4	0.5	14.0	17.7	23.6
4	1	12.9	16.8	26.4
4	2	9.24	10.3	11.3
4	4	5.06	5.94	16.1
4	6	5.53	5.88	6.2
4	SS-0	6.92	5.71	19.2
5	0.25	20.6	22.7	9.7
5	0.5	23.4	28.6	20.1

5	1	23.8	29.9	22.8
5	2	23.5	26.2	10.7
5	4	18.8	21.2	12.1
5	6	15.2	17.5	14.1
5	SS-0	17.9	22.8	24.1
6	0.25	9.49	10.1	6.3
6	0.5	12.7	14.2	11.0
6	1	18.5	18.6	0.4
6	2	22.7	24.6	7.9
6	4	14.4	15.4	6.6
6	6	9.10	7.60	18.0
6	SS-0	7.34	7.23	1.5

SS: single dose at steady-state.

Table S8: Incurred sample reanalysis of the high concentration assay.

Volunteer #	Sampling time [h]	Original analysis [ng/mL]	Reanalysis [ng/mL]	Deviation from mean [%]
1	0.25	6.30	7.39	15.9
1	0.5	9.12	9.68	6.0
1	1	14.7	16.1	8.9
1	2	18.9	20.5	8.2
1	4	11.4	12.9	12.7
1	6	8.20	9.37	13.3
1	SS-0	46.2	49.9	7.8
1	SS-0.25	55.4	60.4	8.7
1	SS-0.5	82.4	87.0	5.4
1	SS-1	150	148	1.1
1	SS-2	217	242	10.8
1	SS-4	316	329	4.0
1	SS-6	294	312	5.9
2	0.25	11.0	11.6	4.6
2	0.5	13.8	14.8	7.2
2	1	15.1	13.9	8.3
2	2	17.4	18.1	3.9
2	4	13.6	13.2	3.2
2	6	9.54	10.1	5.5
2	SS-0	1.56	1.82	15.5
2	SS-0.25	12.4	12.6	1.8
2	SS-0.5	25.9	26.9	3.7
2	SS-1	27.3	29.6	8.2
2	SS-2	55.0	56.5	2.6
2	SS-4	66.1	71.5	7.9
2	SS-6	43.6	43.4	0.3

SS: single dose at steady-state.

Table S9: Incurred sample reanalysis of the low concentration assay.

Volunteer #	Sampling time [h]	Original analysis [ng/mL]	Reanalysis [ng/mL]	Deviation from mean [%]
1	0.25	7.58	7.71	1.7
1	0.5	11.0	11.1	0.7
1	1	16.9	16.5	2.3
1	2	19.9	24.7	21.7
1	4	13.6	14.0	2.7
1	6	10.0	10.9	8.2
1	SS-0	52.5	56.9	8.0
2	0.25	13.2	14.0	6.2
2	0.5	16.6	19.7	17.3
2	1	15.8	15.9	0.9
2	2	19.7	22.1	11.7
2	4	13.0	16.5	23.7
2	6	9.93	12.0	18.9
2	SS-0	1.73	2.07	18.1
4	0.25	14.4	13.8	4.0
4	0.5	17.7	19.5	9.4
4	1	16.8	18.0	6.7
4	2	10.3	13.6	27.2
4	4	5.94	6.66	11.4
4	6	5.88	7.00	17.4
6	0.25	10.1	11.9	16.3
6	0.5	14.2	16.2	13.3
6	1	18.6	24.8	28.7
6	2	24.6	24.2	1.5
6	4	15.4	16.5	7.0
6	6	7.60	12.4	48.0
6	SS-0	7.23	8.87	20.3

SS: single dose at steady-state.

Table S10: Accuracy and precision data for the freeze-and-thaw stability determination.

QC level	Accuracy [%]	Precision [% CV]
QC B	99.6	1.5
QC E	107.7	3.0
QC F	86.5	0.4

N = 3 replicates at each QC concentration.

Table S11: Accuracy and precision data for the 14 days (-20 °C) plasma stability determination.

QC level	Accuracy [%]	Precision [% CV]
QC B	108.7	0.6
QC E	107.2	0.8
QC F	108.8	1.0

N = 3 replicates at each QC concentration.

Table S12: Accuracy and precision data for the 24 h autosampler stability determination.

QC level	Accuracy [%]	Precision [% CV]
QC A	88.8	3.1
QC B	98.3	5.7
QC C	105.9	3.5
QC D	111.8	4.3
QC E	95.9	1.7
QC F	93.1	2.2

N = 3 replicates at each QC concentration.

Table S13: Accuracy and precision data for the 7-week stock solution stability determination.

QC level	Accuracy [%]	Precision [% CV]
QC B	101.6	5.8
QC C	107.5	1.5
QC D	110.2	1.7

N = 3 replicates at each QC concentration.
